# Central nodes of canine functional brain networks are concentrated in the cingulate gyrus

**DOI:** 10.1007/s00429-023-02625-y

**Published:** 2023-03-30

**Authors:** Dóra Szabó, Milán Janosov, Kálmán Czeibert, Márta Gácsi, Enikő Kubinyi

**Affiliations:** 1 Department of Ethology, ELTE Eötvös Loránd University, Budapest, Hungary; 2 Department of Network and Data Science, Central European University, Budapest, Hungary; 3 ELKH-ELTE Comparative Ethology Research Group, Budapest, Hungary; 4 MTA-ELTE Lendület Momentum Companion Animal Research Group, Budapest, Hungary; 5 ELTE NAP Canine Brain Research Group, Budapest, Hungary

**Keywords:** Dog neuroimaging, Resting-state fMRI, Functional connectivity, Network analysis, Graph analysis, Functional brain networks

## Abstract

Compared to the field of human fMRI, knowledge about functional networks in dogs is scarce. In this paper, we present the first anatomically-defined ROI (region of interest) based functional network map of the companion dog brain. We scanned 33 awake dogs in a “task-free condition”. Our trained subjects, similarly to humans, remain willingly motionless during scanning. Our goal is to provide a reference map with a current best estimate for the organisation of the cerebral cortex as measured by functional connectivity. The findings extend a previous spatial ICA (independent component analysis) study (Szabo et al. in Sci Rep 9(1):1.25. 10.1038/s41598-019-51752-2, 2019), with the current study including (1) more subjects and (2) improved scanning protocol to avoid asymmetric lateral distortions. In dogs, similarly to humans (Sacca et al. in J Neurosci Methods. 10.1016/j.jneumeth.2021.109084, 2021), ageing resulted in increasing framewise displacement (i.e. head motion) in the scanner. Despite the inherently different approaches between model-free ICA and model-based ROI, the resulting functional networks show a remarkable similarity. However, in the present study, we did not detect a designated auditory network. Instead, we identified two highly connected, lateralised multi-region networks extending to non-homotropic regions (Sylvian L, Sylvian R), including the respective auditory regions, together with the associative and sensorimotor cortices and the insular cortex. The attention and control networks were not split into two fully separated, dedicated networks. Overall, in dogs, fronto-parietal networks and hubs were less dominant than in humans, with the cingulate gyrus playing a central role. The current manuscript provides the first attempt to map whole-brain functional networks in dogs via a model-based approach.

**Supplementary Information:**

The online version contains supplementary material available at 10.1007/s00429-023-02625-y.

## Introduction

To map similarities and uniqueness of the mammalian brains’ functional connectivity, it is vital to cover a wide array of species from various taxa (Thiebaut de Schotten et al. 2019; Striedter 2002). In this endeavour, species outside of the Primate order are of particular interest, as during Primate evolution, the prefrontal region’s relative size compared to the rest of the cortex increased considerably, which accompanied the differentiation of cortical areas (Wise 2008; Carlén 2017).

Our model, the family dog, a distant taxon, with a population not bred and reared under laboratory conditions but living in its natural social environment, provides a good fit and useful addition to the rapidly increasing field of awake rs-fMRI studies.

The dog brain is currently the focus of several projects, including, among others, EEG (Iotchev et al. 2020), brain banking efforts (Urfer et al. 2021; Sandor et al. 2021, 2022), with fMRI being the most proliferate field in recent years (Aulet et al. 2019; Karl et al. 2021; Prichard et al. 2021). Recent dog fMRI studies are venturing into a wide array of topics, from epilepsy (Beckmann et al. 2021) to comparative face and voice processing (Bunford et al. 2017; Boros et al. 2021; Bunford 2020).

While the default mode network (DMN) is among the most studied human rs-networks, the organisation of the dog DMN is currently disputed in the literature. Although our previous paper described a putative DMN network showing anterior and posterior connectedness, two other research groups reported it to be dissociated to an anterior and posterior hub (Kyathanahally et al. 2015; Beckmann et al. 2020).

In other mammalian species investigated so far (rat Liang et al. 2015, voles Ortiz et al. 2018, ferret Zhou et al. 2016, marmoset Belcher et al. 2013 and macaques Hutchison et al. 2011), the putative DMN contained both the posterior cingulate cortex and frontal cortical areas.

Our main goal was to create a map of functional brain organisation for a non-primate species with well-studied, complex cognition and social behaviour (Miklosi and Kubinyi 2016) in similar detail and approach to that suggested by recent papers (Thiebaut de Schotten et al. 2019; Keifer and Summers 2016; van den Heuvel et al. 2016).

Comparative connectomics (van den Heuvel et al. 2016) aims to understand the common architectures and species-specific differences of brain topology. To achieve this, we need to reconstruct connectomes of different species suitable for such comparisons.

In our previous paper (Szabo et al. 2019) we described a functional connectivity-based parcellation of the dog brain. The main reason we had to rely on a model-free approach was the lack of published dog brain atlases with voxel-level anatomical labelling. Since then, several new stereotaxic atlases have been published with better spatial resolution and more detailed parcellation (Czeibert et al. 2019; Johnson et al. 2020), including probability tissue masks for white and grey matter (Liu et al. 2020).

While our previous approach allowed us to describe the spatial characteristics of the networks without the selection of a priori seed regions, it was limited by the lack of anatomically bound, standardised ROIs, which makes comparisons across studies and species difficult. The ICA approach is less suited to provide information about the functional organisation beyond certain voxels belonging to the same, statistically independent source signal (Beckmann 2012). A model-based approach can reveal the network of regions with the strongest functional connections to the respective ROI (Van Den Heuvel and Pol 2010) and inform us about the strength of individual intra- and inter-network connections via semi-partial correlations. Semi-partial correlations indicate a unique relationship between an independent and the dependent variable (ROI time series), where the variance in a dependent variable is explained only by that independent variable, excluding the variance explained by other independent variables.

In our current study, we utilise ROI based approach and analyse the resulting undirected graph to group ROIs into networks to describe their characteristics and determine the most central nodes of the resulting graph. We also aimed to compare the outcome of the two approaches to understand their impact.

## Materials and methods

### Subjects

We measured 33 family dogs *(Canis familiaris)* (age 6.70 ± 3.62 years (mean $\pm$ SD), range 1–14 years, 17 females and 16 males, 9 border collies, 7 golden retrievers, 5 mongrels, 2 Australian shepherd, 1 English cocker spaniels, 1 Labrador retriever, 1 labradoodle, 1 mudi, 1 Swiss shepherd dog, 1 Tervueren, 1 springer spaniel, 1 Chinese crested dog, 1 Cairn terrier, 1 Hungarian vizsla). The training procedure has been described in detail in a previous study (Andics et al. 2014) and was based on individual and social learning using positive reinforcement.

### Procedure

**Fig. 1:**
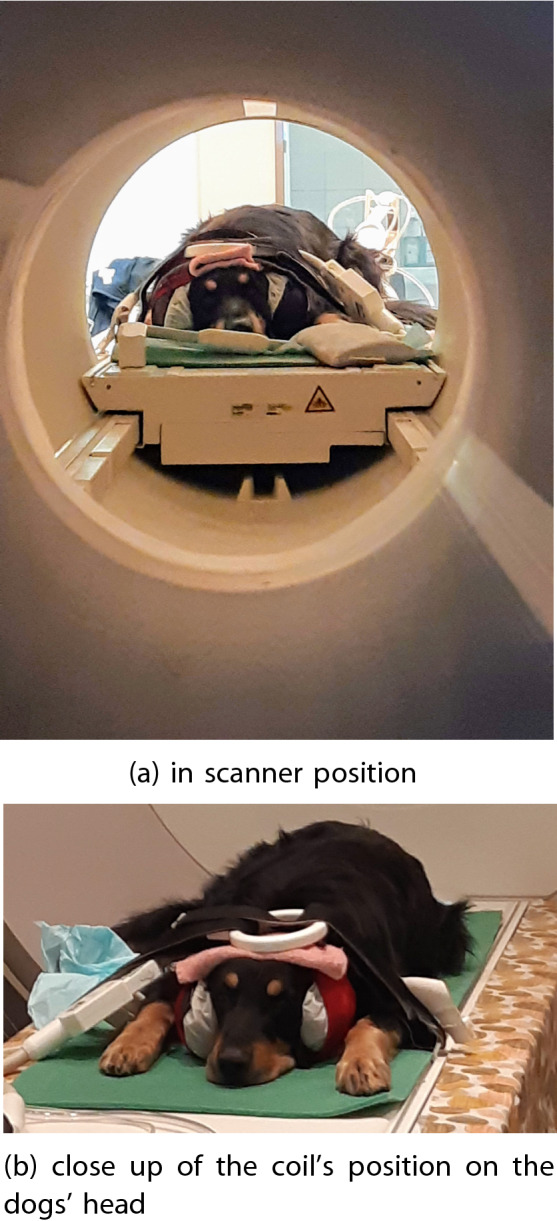
Dogs in scanner positioning

The study consisted of a 2-minute-long habituation phase to familiarise the dogs with the semi-continuous scanning procedure, followed by two 6-minute-long data collection runs. To provide sound protection, the dogs were wearing ear muffs. During scanning, dogs were lying with their eyes open, without presenting a fixation cross, with their handler visible, but avoiding eye contact with the subject. Figure 1a shows the dogs’ position in the scanner. The strap over the head was used to fixate the circular coil on the top of the dog’s head, not to restrain dog motion (Fig. 1b). Dogs were not fixed (could leave their position at any time) or sedated in any way. The motion threshold for successful runs was set to a maximum of 3 mm (for each translation direction) and 2 degrees (for each rotation direction) during the whole run.

### Ethics statement

All procedures were approved by the Ethical Committee of Eötvös Loránd University (KA-1719/PEI/001/1490-4/2015) and by the Government Office of Pest County Directorate of Food Chain Safety and Animal Health (XIV-I-001/520-4/2012) and conducted in accordance with relevant guidelines and regulations. Owners provided informed consent and were able to stop the procedure at any time.

### Image acquisition

Functional MRI acquisitions were performed on a Siemens Prisma 3T scanner Siemens Healthcare, Erlangen, Germany) using a Gradient Echo Echo Planar Imaging (GRE-EPI) sequence with TR = 2640 ms, including a 500-ms delay at the end of each volume, and TE = 31 ms. We decided to introduce the delay for welfare reasons. During piloting, we realised that our dogs seemed to find continuous scanning less comfortable than protocols with short gaps between subsequent scans. The 500 ms delay was selected empirically during piloting to include the shortest gaps that the dogs are still comfortable with. The protocol had an in-plane Field-of-View 128 mm $\times$ 128 mm, using 2 mm in-plane resolution and 2 mm slice thickness, measuring 28 slices with an inter-slice gap of 0.5 mm, using an excitation flip angle of $86^{\circ }$. Phase-encoding direction was set to foot $\gg$ head. A single loop coil ($\hbox {d}=11$ cm) was used for signal detection, fixed to the table of the scanner. These straps kept the coil in position on the head of the dog. The dogs were able to pull out their heads from these straps. In this case, the headphones would have remained still on their head as that was fastened with a separate layer. This event occurred only during the training sessions.

In each run, 137 volumes were acquired, with the first five discarded before processing, resulting in a total functional scanning time of 362 s/run. A T1-weighted anatomical scan was carried out separately as part of another study on each awake dog for spatial registration on a 3T Philips Ingenia scanner (Philips Medical Systems, Best, The Netherlands), using a 3D Turbo Field Echo (TFE) sequence, with TR = 9.85 ms, TE = 4.6 ms, and an isotropic resolution of 1 mm.

### Data analysis

FMRI preprocessing relied on SPM12 (http://www.fil.ion.ucl.ac.uk/spm/) and CONN (Alfonso Nieto-Castanon 2020). The process included the following steps: affine realignment (6 parameters, least square approach), reslicing, manual coregistration of the mean image to the individuals’ own structural T1 image in Amira 6.0 (Thermo Fisher Scientific), normalisation of the functional images to this transformed mean functional image via SPM’s standard nonlinear warping function with 16 iterations.

The following steps were carried out in CONN software: 1. functional outlier detection (ART-based identification of outliers scans for scrubbing), 2. segmentation of CSF, WM, GM (Liu et al. 2020), 3. regressing out confounding effects, 4. functional smoothing (spatial convolution with 4 mm Gaussian kernel), 5. denoising with bandwidth filtering (0.001 Hz–0.1 Hz), 6. linear detrending.

CONN’s default denoising pipeline implements an anatomical component-based noise correction procedure (aCompCor), which estimates and removes confounding effects separately for each voxel/run/subject using Ordinary Least Squares (OLS) regression to project each BOLD signal time series to the sub-space orthogonal to all potential confounding effects. The pipeline covers noise components from WM and CSF, estimated subject-motion parameters, identified outlier scans or scrubbing, constant and first-order linear session effects (Alfonso Nieto-Castanon 2020).

The analysis was carried out in the BBT stereotaxic template space (Liu et al. 2020). We relied on the ROI segmentation of (Liu et al. 2020), apart from the cingulate gyrus, where we used the more detailed segmentation provided by Johnson et al. (2020). We renormalised these ROIs to the BBT. This approach assigns hubs/nodes of functional interests based on myeloarchitectonic-based histology atlases. The analysis space was set via an explicit brain mask from a liberal brain template and was relying on percent signal change instead of the bold signal raw value.

From the available 111 ROIs, we analysed only the 97 cortical and sub-cortical ROIs. We decided to exclude ROIs belonging to the brainstem and olfactory regions to focus our analysis on the cortical functional brain organisation. The list of the excluded ROIs can be found in the appendix.

### Statistical analysis

To evaluate the similarities and differences between the resting state network organisation described in our current and previous paper (Szabo et al. 2019), we calculated ROI-based Jaccard similarity.

To obtain an ICA network to ROI mapping, we relied on the ICA mask files containing the results of our previous paper (Szabo et al. 2019), normalised it into the current atlas space and projected it over the ROI atlas. For each network, we extracted the number of overlapping voxels with each ROI. Due to the nature of the ICA approach, several networks involved the same ROIs, therefore, we allocated each ROI to the network with the largest overlapping volume/majority vote. In the case of bilateral ROIs, we averaged the number of overlapping voxels for the two ROIs for each ICA network before allocation to ensure we allocate bilateral ROIs to the same network.

In the current study, we analysed seed-based connectivity (“Does the average connectivity between ROIs differ from zero?”). For first-level functional connectivity maps, semi-partial correlation and weighted GLMs were calculated. For generating the reported 2nd level connectivity map results, we relied on applied parametric multivariate statistics with the following post hoc thresholds (Benjamini and Hochberg 1995): cluster threshold p-FDR corrected$<0.05$ (MVPA omnibus test) and connection threshold p uncorrected$<0.01$.

This approach addresses the multiple comparison problems inherent to this type of analysis and is suited to handle the high rates of false positives. It calculates weighted graphs representing the level of effective or direct connectivity between an individual ROI and every other analysed ROI in the brain after discounting effects that may be mediated or accounted for by other ROIs.

We transformed the ROI matrix into a network and analysed it by using Python and the NetworkX (Hagberg et al. 2008) library. In this weighted undirected network, every node corresponds to a brain region, while the strength of their connection is proportional to their ROI values.

We captured the so-called small-worldness property, which quantifies how well the neighbouring node’s neighbours are connected—how easily different nodes can reach other nodes within the network (Watts and Strogatz 1998). We did so by computing the classical small-world scalar ($\Sigma$) (Humphries et al. 2006), normalised clustering coefficient ($\Gamma$) and normalised path length ($\Lambda$) (Bassett and Bullmore 2017).

Next, we extracted network communities—network subgraphs whose’ nodes are densely interconnected compared to the rest of the network—by following widely-used methodologies (Lambiotte et al. 2008).

Finally, we visualised and explored the network communities by using Gephi 0.9.2 (Bastian et al. 2009) with two layers. Firstly, we plotted all analysed ROIs (except cerebellum) with various modularity thresholds to find the optimal value empirically. This gave us the initial structure with 10 modules (with a modularity threshold of 0.6). Then we ran 10 repeats to produce a consensus voting for this structure. For the second layer, we took the most populated two lateralised modules and plotted these two in a separate round, which resulted in the final, combined 12 (8+4) module structure.

## Results and short discussion

### ROI to ROI analysis

**Fig. 2:**
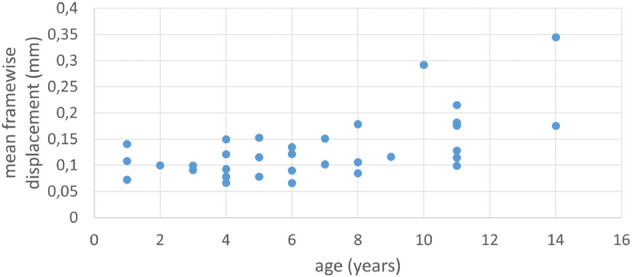
Correlation between age of subjects and mean FD

Mean (scan-to-scan) motion was 0.1314 ± 0.1340 mm $(mean \pm SD)$. There was a moderate positive correlation between the subject’s age and mean framewise displacement (FD) value (${\rho}=0.52$, ${p}<0.005$) (Fig. 2).

Due to the significant correlation between age and FD, we decided not to look for any relationship between age and connectivity, as our sample size was not suited to tackle the significant correlation (even though all subjects did pass the motion threshold determined for a successful run and were analysed). Recent research shows that normal ageing produces a significant increase in head motion parameters, therefore, this relationship is not unexpected (Sacca et al. 2021).

While cerebellar regions were included in the data, no connection survived post hoc corrections, hence they are not reported and listed in the visuals. In our previous publication, the cerebellar networks also showed up as separate, isolated network (Szabo et al. 2019). Another possible reason for the lack of such connections can be the exclusion of brainstem ROIs from the current analysis.

**Fig. 3:**
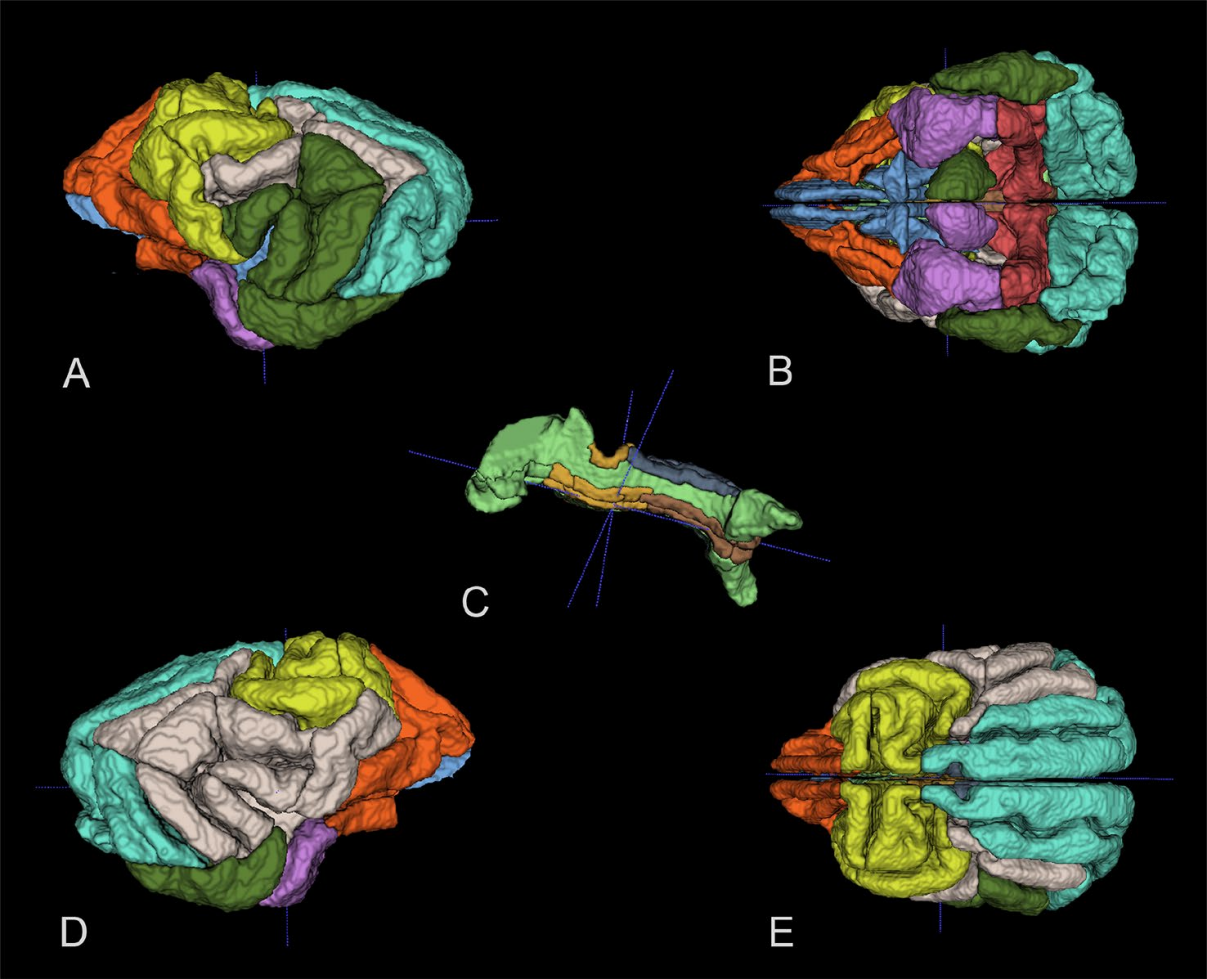
Selected views of the functional brain networks overlaid on 3D stereotaxic brain atlas. Different colours represent different functional networks. **A** Left, **B** Ventral, **C** Extracted cingulate gyrus region, **D** Right, **E** Dorsal

An overview of the resulting parcellation can be found in Fig. 3

### Similarities to previous dog ICA rs-networks

Despite the inherently different approach of ICA and ROI-based analysis, the resulting networks showed a remarkable similarity (Table 1), with Jaccard similarity values between 0.64–0.80. This means that the majority of the ROIs were part of the corresponding networks in our previous study (Szabo et al. 2019) as well.

However, unlike in our previous study, we could not detect a clear, distinct auditory network candidate. Instead, we had two lateralised networks (Sylvian L, Sylvian R), including the respective Sylvian (auditory) regions among other regions (Fig. 3). Possible explanations include insufficient parcellation (current stereotaxic atlases segment this gyrus into three subregions, but these subjectively drawn regional boundaries lack clearly defined and constant anatomical margins) next to individual variation in gyrification and functional boundaries. For the reliable detection of auditory regions, individual, voxel-based localisation is currently a better approach in dogs, as they are not following anatomic boundaries, and are located in a small region containing several functions, without sub-millimetre resolution being feasible in awake dog fMRI. Future work is needed to further decrease the uncertainty regarding functional boundaries in dog auditory cortex, to functionally delineate A1 and subsidiary functional auditory regions with a large sample size, accompanied with cytoarchitecture work and tracing to establish a clearly anatomically defined extent of auditory regions and their anatomical connectivity in the dog. The two, extensive, lateralised functional networks appear in both studies despite the different phase encoding directions and analytical approaches.

While we reported the anterior and posterior dog default mode network (DMN) to show functional connectedness in the previous study, the connection between them in the current seed-based analysis is less convincing. Although we found connections between the splenial gyrus and anterior cingulate regions (XMII, GI), the splenial gyrus was listed as part of the Visual network, not as part of the Ventral posterior cingulate network. A more detailed segmentation of this region in future atlases may reveal that this area plays a role in multiple networks. Other recent publications found no sign of antero-posterior connectedness of the DMN in the dog brain via rs-fMRI. The insufficient resolution, susceptibility artefact, cross-subject variations (Bijsterbosch et al. 2018), or possible breed/skull shape-related differences are all possible alternative explanations which need to be excluded before we can determine whether the dog DMN is indeed dissociated. Previous dog tractography studies aimed specifically to describe the structural basis of specifically the dog DMN (Robinson et al. 2016) and whole dog brain (Jacqmot et al. 2013). Robinson et al. (2016) reported that the antero-posterior tract between putative aDMN and pDMN regions was found only in 9 out of 23 dogs which, unlike in humans, points toward a significant individual variability in dogs. Further structural connectome studies obtained with tracing and optical imaging are necessary to uncover the underlying reasons responsible for this divergence.

**Table 1:** Comparing the resulting resting state network organisation across the two approaches

ICA based network (Szabo et al. 2019)	Taxonomy (Uddin et al. 2019)	ROI based network	Jaccard index
A	M-FPN	Hippocampal	0.66
		Ventral posterior cingulate	
B	M-FPN	Medial prefrontal	0.64
C	D-FPN/L-FPN	Dorsal/lateral prefrontal	
D	M-CIN	Amygdala	0.71
		Partial Sylvian L	
E	M-CIN	Partial Sylvian R	
F	PN	Partial Sylvian R (Sensorimotor)	
I	PN	Partial Sylvian L & partial Sylvian R (Auditory)	
J	M-CIN	Mid-cingulate	
K	PN	Sensoritomotor (peri-cruciate)	0.74
	M-CIN	Anterior cingulate	
H	M-FPN	Dorsal posterior cingulate	0.80
L	ON	Visual	0.75
M	ON		

### Network characteristics

#### Small world

**Fig. 4:**
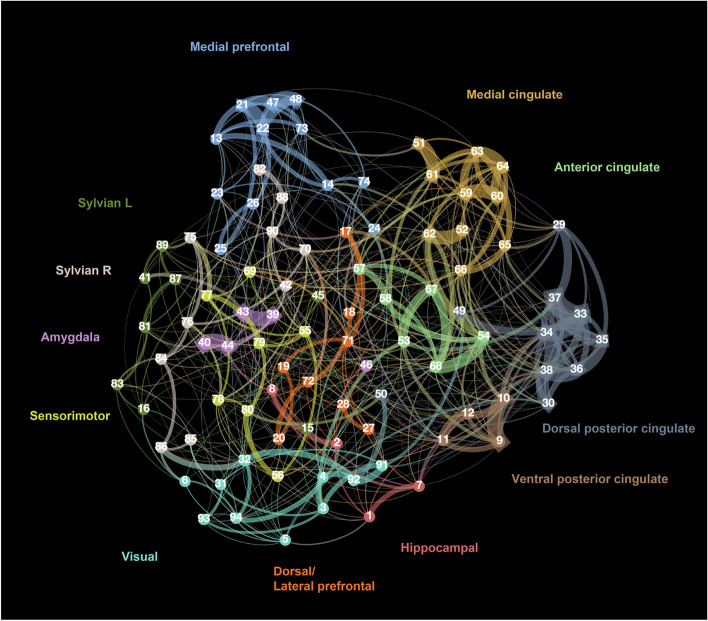
The organisation of the functional network nodes in the dog brain visualised by Gephi using ForceAtlas layout. The ForceAtlas layout simulates a physical system consisting of particles linked by different attractive and repulsive forces that organize the network into an organic topology reflecting the general connectivity patterns. Node and edge colouring correspond to community membership, while the edge widths measure the semi-partial correlation values. The list of numbered nodes can be found in the supplementary material, while the main brain regions are labelled with appropriate colours

The analysed network consisted of 97 nodes and 560 edges, containing 427 triangles (Fig. 4). The connection density of the resulting network was 12%. This is within the reported range of fMRI connectivity studies in other species (5% to 30%) (Bassett and Bullmore 2017), reflecting that the applied strict threshold was adequate to account for the high level of noise inherent in rs-fMRI data (Tables 2, 3).

**Table 2:** Overall network properties of the dog brain

Variable	Symbol	Normalised value	Raw value
Normalised clustering coefficient	Gamma ($\Gamma$)	1.45	0.18
Normalised path length	Lambda ($\Lambda$)	1.02	2.15
Classical small-world scalar	Sigma ($\Sigma$)	1.42	NA

These network characteristic values are also similar to those reported in Bassett and Bullmore (2017) for macaque and mouse brain networks. Small-world networks have normalised clustering coefficient $> 1$, normalised clustering coefficient $\approx 1$ and classical small-world scalar $> 1$Humphries et al. (2006), meaning the dog brain network satisfies the requirements of a small-world network.

### Modularity

For bilateral homologous regions, both node degree (${rho}=0.49$, *p* $< 0.001$) and node weight (${rho}=0.87$, *p* $< 0.001$) showed highly significant positive correlations.

We looked at modularity structure on two levels. First, we included all analysed ROIs, which resulted in 10 modules.

**Fig. 5:**
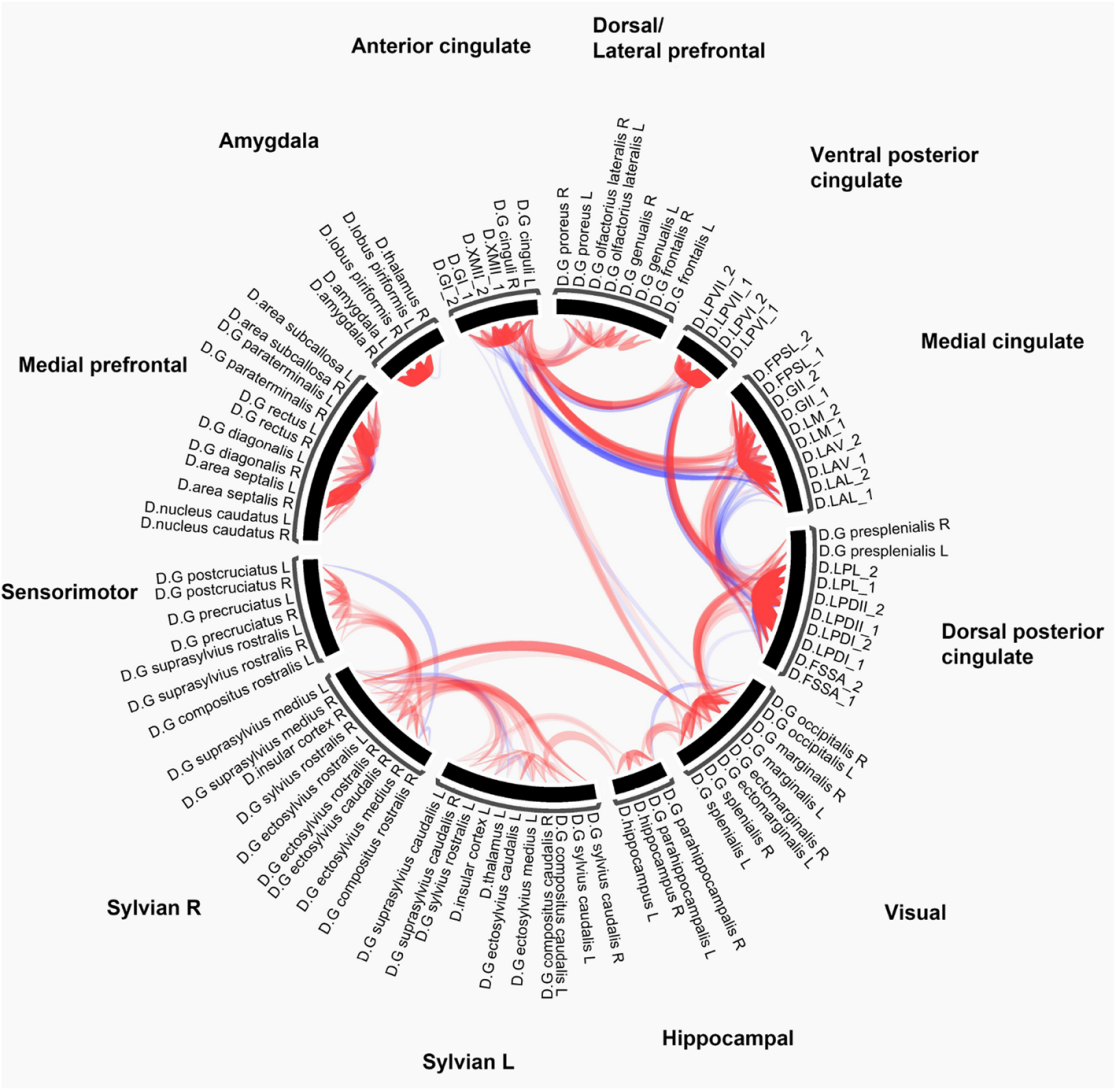
Colorwheel visualisation of positive (red) and negative (blue) ROI to ROI connections. Width and opacity of edges proportional to the strength of the connection

Out of these, the two largest communities were lateralized, consisted of 15–15 ROIs and covered most of the temporal and parietal surface of the brain. In these two entities, insular, sylvian, ectosylvian, rostral composite regions got split based on laterality (Fig. 5).

Further lowering modularity resolution did not result in breaking up these two large groups. Therefore, we decided to carry out a second analysis restricted to the nodes of these two entities to understand their functional organisation in more detail. This resulted in dividing each into two further communities, therefore the final segmentation contained 12 modules in total (Fig. 3).

**Fig. 6:**
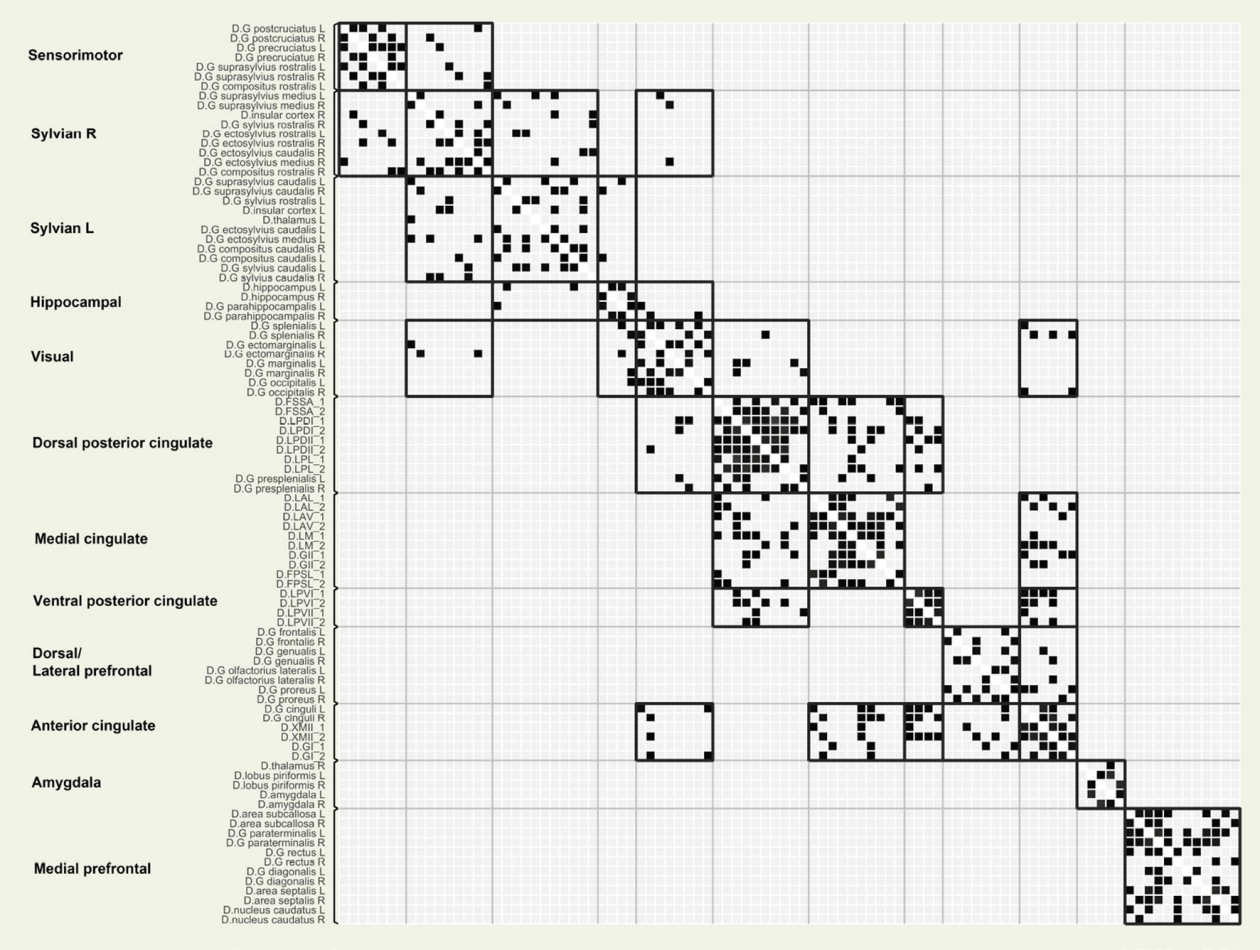
Thresholded semipartial correlation matrix of the ROI to ROI connections. Only significant connections are displayed. P values p-FDR corrected$<0.05$ at the cluster level (MVPA omnibus test), connection threshold p values uncorrected$<0.01$

**Table 3:** Main regions involved and taxonomy of the brain network types based on Uddin et al. (2019)

Name	Network type	Main regions/gyri involved
Visual	ON	Splenialis, ectomarginalis, marginalis, occipitalis and suprasylvius caud.
Dorsal/Lateral prefrontal	D-FPN/L-FPN	Genualis, olfactorius lateralis, frontalis and proreus
Medial prefrontal	M-FPM	Area subcallosa, paraterminalis, rectus, diagonalis, area septalis, nucleus caudatus
Hippocampal	M-FPM	Hippocampus, parahippocampalis
Ventral posterior cingulate	M-FPM	LPVI, LPVII
Dorsal posterior cingulate	M-FPM	FSSA, LPDI, LPDII, LPL, presplenialis
Mid-cingulate	M-CIN	FPSL, GII, LAL, LAV, LM
Anterior cingulate	M-CIN	GI, XMII, remaining cinguli
Amygdala	M-CIN	Amygdala, lobus piriformis
Sensoriomotor	PN	Pre- and postcruciatus, suprasylvius rostr
Sylvian L	PN	(Ventral post anchored) comp. caud., L regions
Sylvian R	PN	(Dorsal anchored) ectosylvius rost., suprasylvius medius, R regions

#### Density within communities

All resulting network communities (subgraphs) had a significantly higher edge density than the whole network (Table 4), meaning that in a network community, any pairs of nodes are more likely to be connected to each other than to any other node not part of that community. The densest network communities only contained ROIs from the cingulate gyrus (ventral posterior cingulate, dorsal posterior cingulate, anterior cingulate, middle cingulate), showing the core role of this region within the dog brain (Fig. 6).

**Table 4:** Number of nodes and density values within network communities for the respective functional networks

Network	No. of nodes	Density
Whole	97	0.12
Visual	8	0.47
Hippocampal	4	0.5
Ventral posterior cingulate	4	0.75
Dorsal/lateral prefrontal	8	0.34
Dorsal posterior cingulate	10	0.62
Medial prefrontal	12	0.46
Anterior cingulate	6	0.55
Mid-cingulate	10	0.52
Amygdala	5	0.4
Sensoriomotor	7	0.49
Sylvian L	10	0.34
Sylvian R	10	0.3

### Most central nodes and edges

We present the top nodes listed by weighted degree, degree and betweenness centrality values. In the case of bilateral regions, we calculated the average of the two nodes.

Ten of these ROIs were ranked within the top 20 for all three categories (Table 5): area subcallosa, prorean gyrus, splenial gyrus, posterior cingulate gyrus (LPDI, LPVI, LPVII), anterior cingulate gyrus (GI, XMII, G cinguli) and mid-cingulate gyrus (LM).

**Table 5:** Most central nodes by weighted degree (WD), degree (D) and betweenness centrality (BC) values. Top 20 bilateral nodes listed in each ranking. Bilateral values were calculated by averaging the two nodes prior to ranking

ROI	WD	ROI	D	ROI	BC
LPDI	101.15	LPDI	19	LM	121.19
LPL	92.37	LM	19	g. cinguli	107.07
LPDII	76.38	G. cinguli	18	g. proreus	90.8
LPVI	76.38	LPVI	16.5	LPDI	83.6
g. cinguli	71.93	XMII	16	LPVI	82.55
LAV	65.39	LPL	14.5	Area subcallosa	82.3
XMII	64.17	LPDII	14.5	G. suprasylvius rostralis	81.6
LM	62.2	FSSA	14.5	lobus piriformis	78.17
G. paraterminalis	60.25	LPVII	14.5	LPVII	76.82
LAL	56.44	LAV	14.5	XMII	75.36
FSSA	56.27	Area subcallosa	13.5	G. precruciatus	75.19
GII	44.35	GII	13	G. ectomarginalis	74.88
LPVII	53.55	G. suprasylvius rostr	13	G. suprasylvius caud	72.04
Area septalis	50.99	G. proreus	13	G. compositus rostr	71.92
Area subcallosa	46.4	GI	12.5	G. splenialis	69.83
G. splenialis	45.93	LAL	12.5	GI	69.3
l piriformis	44.74	G. splenialis	12.5	G. ectosylvius caud	63.44
GI	44.34	G. precruciatus	12.5	G. compositus caudalis	62.82
G. proreus	41.59	G. ectomarginalis	12.5	G. sylvius rostr	59.41
G. marginalis	40.98	G. compositus rostr	12	G. rectus	58.32

7 of the 10 most central nodes are part of the cingulate gyrus, while all 7 nodes belong to separate networks.

There were two networks without inter-network connections (with a direct link stronger than our threshold), the amygdala network and the medial prefrontal network (Fig. 5). We found no direct links between the anterior cingulate and dorsal posterior cingulate gyrus, nor between the ventral posterior cingulate and mid-cingulate areas, which would have been our expectation for a direct aDMN and pDMN connection. This does not mean that these networks are functionally isolated from other parts of the brain. The lack of direct links surviving the current threshold could be the result of mediation by other regions, because the utilised semi-partial correlation only shows the unique relationship between two ROIs, excluding variance shared with other independent variables.

The two lateralised Sylvian networks were highly connected. Next to covering associative and sensorimotor cortices, these networks also included the auditory regions and the insular cortex. The dorsal/lateral prefrontal network was only connected to the anterior cingulate network. The anterior cingulate network also had links to the ventral posterior cingulate, mid-cingulate, dorsal/lateral prefrontal and the visual network.

## Conclusions

We scanned awake dogs in a “task-free condition”, a cognitive state hard to define objectively (Gorges et al. 2017), especially in the case of animals where scanner fixation is widely used (e.g. rabbits Weiss et al. 2018). Our subjects, similarly to humans, remain willingly motionless during scanning. Recently, a condition called “naturalistic viewing” (no fixation-cross, high in social content, evoking variable gaze positions) was found to be outperforming the standard resting condition in humans both in regard to functional connectivity-based prediction of behaviour (Finn and Bandettini 2021) and test-retest reliability (Wang et al. 2017). This supports our claim that the free-viewing and fixation-free condition we measured the dogs in provides relevant data.

We found that even in the case of successful sessions (defined by session-level total motion thresholds), there is a significant correlation between the age of the subject and framewise displacement. This means that in dogs, similarly to humans (Sacca et al. 2021), normal ageing results in increasing head motion. Differences between the age groups’ motion levels have to be considered and adequately addressed if the goal is to interpret differences in functional connectivity networks across such groups. Future studies (apart from collecting data from more subjects) may try to utilise special, extra preprocessing steps which address this in humans, for example, via taking into account framewise displacement and DVARS during scrubbing as proposed by Power et al. (2012). Due to the currently available sample size, we were unable to investigate the putative effects of age or breed differences on the organisation of functional networks. Future multi-site studies with standardised scanning protocols are more likely to achieve the required sample size for such endeavours.

Due to the susceptibility artefact around the sinuses, we decided to exclude olfactory regions from our analysis. The level of noise and signal loss makes data from this area inaccessible/unreliable with the current hardware and coil. This prohibited the exploration of a possible olfactory network and could contribute to the list of reasons why we did not detect a clear antero-posterior DMN connectivity (although we found connections between the splenial gyrus and anterior cingulate regions), as in our previous study, we did not exclude these regions from the analysis.

The existence of the antero-posterior DMN connectivity in dogs is debated, with several studies not finding supporting evidence for it Kyathanahally et al. (2015), Robinson et al. (2016), Beckmann et al. (2020). Given that the current result is also inconclusive in this regard (strong individual ROI to ROI connections between the respective networks were present, but no connection on the network level surviving post hoc corrections), we do not feel we can make a binary conclusion regarding the definitive presence or absence of this connection based on the currently available information.

The currently described network organisation closely resembles our previous findings, despite the different approaches (model-free vs. model-based) between the two studies.

The human lateral prefrontal cortex (LPFC) is disproportionately expanded even when compared to non-human primates (Vendetti and Bunge 2014). In dogs, on the other hand, it is a small area, including less than 10% of the cortical regions. It consists of mostly the frontal and prorean gyrus, which have a combined total volume of c.a 400 voxels/3200 cm^3^. This area also suffers from susceptibility artefact/signal loss due to the extensive sinuses.

The human LPFC, thought to be related to relational reasoning (Vendetti and Bunge 2014) plays a part in three functional network groups (D-FPN, L-FPN, M-CIN) (Uddin et al. 2019). In contrast, the whole dog LPFC belongs to a single resting state network (Dorsal/lateral prefrontal). The lack of separate attention and control networks in dogs could be a byproduct of the limits of the currently available image resolution, which can be further improved by developing coils specifically tailored to measure dogs (Guran et al. 2022). Alternatively, in dogs, the cingulate cortex may be handling some integrative functions which were taken over by frontoparietal regions during primate brain evolution.

The cingulate cortex has been consistently identified as a hub with a high level of centrality, as well as dense and widespread connectivity in comparative studies across humans, chimpanzees, macaques, and cats (van den Heuvel and Sporns 2013). The human posterior cingulate gyrus shows high node degree and betweenness centrality, suggesting it is essential for integrating and passing information across networks. In macaques, the posterior cingulate gyrus showed the highest node degree and betweenness centrality values (Miranda-Dominguez et al. 2014). Our results regarding the centrality of the cingulate cortex in dogs are in line with these findings. Areas across the anterior (GI, XMII), medial (LM) and posterior (LPDI, LPVI, LPVII) cingulate gyrus are all found among the most central nodes, next to the prorean gyrus and splenial gyrus. Functional dog fMRI studies also report the involvement of the cingulate gyrus across several tasks, such as word detection and odour discrimination (Prichard et al. 2018, 2020)

The cingulate cortex plays a central role in various functions related to behavioural flexibility, ranging from autonomic regulation to action selection and performance monitoring. It is a processing hub for the regulation of autonomic responses, assessing emotional and motivational aspects of internal and external information (seeing emotional faces or listening to emotionally charged voices), cognitive control, such as response selection, attentional processing, monitoring conflict, detecting errors, and reward-based decision-making (van Heukelum et al. 2020).

Dogs’ cingulate cortex is relatively larger than in humans when compared to the total cortex volume. Functional studies testing whether and where in the dog cingulate cortex all the above-mentioned processes are localised, or determining the level of frontoparietal integration in these tasks in dogs are yet to be carried out. Future studies should also include validated cytoarchitecture work and extensive tracing to allow us to establish a clearly anatomically defined extent of cingulate and its anatomical connectivity in the dog. This is a prerequisite for a more detailed parcellation of the splenial and cingulate gyrus, which in turn is necessary to determine whether e.g., the cingulate gyrus is handling integrative functions that were taken over by frontoparietal regions in the case of the human brain.

To overcome some disadvantages of the model-blind approach utilised in our previous paper, the current research set out to describe the functional organisation via a model-driven analysis. This allowed us to look at both inter- and intra-network connections, describe the most central nodes, and evaluate the whole brain as a single large network while providing more straightforward interpretability,

Both approaches have advantages and disadvantages, focusing on a different aspect of brain networks, yielding similar, but not identical network structures. Neither is able to provide definitive answers, or certainty about the functional organisation of the dog brain, as both are mere models and approximations. By applying the model-based approach, we adhere to how the first layers of knowledge were built up at the early stage of human functional brain mapping, as early human rs-fMRI studies typically characterised functional connectivity via a small number of large-scale spatial maps as well Smith et al. (2013).

We relied on the universal resting state network taxonomy proposed by Uddin et al. (2019) to categorise dog networks. Currently, compared to the field of human fMRI, we have very few studies describing the functions of respective brain areas in dogs. Therefore, we had to resort to anatomical and evolutionary parallels where the functional parallels are yet untested. We found examples for most major network types and that anatomical modules correspond to groups/communities of functional networks in the dog brain.

Stimulus-based fMRI studies are needed to assign functions to the networks described in this paper. The current reference map allows researchers to carry out future connectivity fingerprint matching studies (Thiebaut de Schotten et al. 2019) across even more species, now including the dog as well.

### Supplementary Information

Below is the link to the electronic supplementary material.

Supplementary file 1 (PDF 97KB)

## Data Availability

The datasets generated and analysed during the current study are available from the corresponding author on reasonable request.

## Appendix

Supplementary table including number codes of ROIs.

List of excluded ROIs:

bulbus olfactorius L

bulbus olfactorius R

commissura rostralis

diencephalon

hypophysis

medulla oblongata

medulla spinalis

mesencephalon

nervus opticus

pedunculus olfactorius L

pedunculus olfactorius R

pons

tuberculum olfactorium L

tuberculum olfactorium R

hemispherium cerebelli L*

hemispherium cerebelli R*

vermis cerebelli*

*included in the analysis, but not shown on the figures due to a lack of inter-network connection above the reporting threshold
