# Supplementary material for: Central nodes of canine functional brain networks are concentrated in the cingulate gyrus
Source: Brain Struct Funct. 2023 Mar 30;228(3-4):831–43. doi: 10.1007/s00429-023-02625-y (PMC10147816; doi:10.1007/s00429-023-02625-y)
Supplement: Supplementary file 1 — (PDF 97KB) [file 429_2023_2625_MOESM1_ESM.pdf]

Supplementary material

Central nodes of canine functional brain networks are concentrated in the cingulate gyrus

Dóra Szabó[1], Milán Janosov[2], Kálmán Czeibert[1], Márta Gácsi[1,3], Enikő Kubinyi[1,4,5]

[1] Department of Ethology, ELTE Eötvös Loránd University, Budapest, Hungary
[2] Department of Network and Data Science,Central European University, Budapest, Hungary
[3] ELKH-ELTE Comparative Ethology Research Group, Budapest, Hungary
[4] MTA-ELTE Lendület Momentum Companion Animal Research Group, Budapest, Hungary
[5] ELTE NAP Canine Brain Research Group, Budapest, Hungary

E-mail: szaboodoora@gmail.com, eniko.kubinyi@ttk.elte.hu

Table S1. List of ROIs, the 97 nodes of the analysed cortical and sub-cortical networks (see Fig. 4 in the main text), and their respective hex colour codes. (The hex colour code is a 6-symbol code made of up to three 2-symbol elements. Each of the 2-symbol elements expresses a colour value from 0 to 255.) L: left, R: right

ROI name	Code	Network	Hex code
G. parahippocampalis L	1	Hippocampal	#d06465
G. parahippocampalis R	2	Hippocampal	#d06465
G. splenialis L	3	Visual	#79d8c5
G. splenialis R	4	Visual	#79d8c6
G. suprasylvius caudalis L	5	Visual	#79d8c7
G. suprasylvius caudalis R	6	Visual	#79d8c8
Hippocampus L	6	Hippocampal	#d06465
Hippocampus R	8	Hippocampal	#d06465
LPVI_1 Limbica posterior ventralis L	9	Ventral posterior cingulate	#a37b5d
LPVI_2 Limbica posterior ventralis R	10	Ventral posterior cingulate	#a37b5d
LPVII_1 Limbica posterior ventralis II L	11	Ventral posterior cingulate	#a37b5d
LPVII_2 Limbica posterior ventralis II R	12	Ventral posterior cingulate	#a37b5d
Area subcallosa L	13	Medial prefrontal	#7ba5d10
Area subcallosa R	14	Medial prefrontal	#7ba5d10

ROI name	Code	Network	Hex code
G. compositus caudalis L	15	Sylvian L	#698838
G. compositus caudalis R	16	Sylvian L	#698839
G. genualis L	17	Dorsal/lateral prefontal	#e57027
G. genualis R	18	Dorsal/lateral prefontal	#e57027
G. olfactorius lateralis L	19	Dorsal/lateral prefontal	#e57029
G. olfactorius lateralis R	20	Dorsal/lateral prefontal	#e57030
G. paraterminalis L	21	Medial prefrontal	#7ba5d10
G. paraterminalis R	22	Medial prefrontal	#7ba5d10
G. rectus L	23	Medial prefrontal	#7ba5d10
G. rectus R	24	Medial prefrontal	#7ba5d10
G. diagonalis L	25	Medial prefrontal	#7ba5d10
G. diagonalis R	26	Medial prefrontal	#7ba5d10
G. frontalis L	27	Dorsal/lateral prefontal	#e57031
G. frontalis R	28	Dorsal/lateral prefontal	#e57032
FSSA_1 Fissura suprasylvalis anterior L	29	Dorsal posterior cingulate	#6c7b8d
FSSA_2 Fissura suprasylvalis anterior R	30	Dorsal posterior cingulate	#6c7b8d
G. ectomarginalis L	31	Visual	#79d8c9
G. ectomarginalis R	32	Visual	#79d8c10
LPDI_1 Limbica posterior dorsalis L	33	Dorsal posterior cingulate	#6c7b8d
LPDI_2 Limbica posterior dorsalis R	34	Dorsal posterior cingulate	#6c7b8d
LPDII_1 Limbica posterior dorsalis II L	35	Dorsal posterior cingulate	#6c7b8d
LPDII_2 Limbica posterior dorsalis II R	36	Dorsal posterior cingulate	#6c7b8d
LPL_1 Limbica posterior lateralis L	37	Dorsal posterior cingulate	#6c7b8d
LPL_2 Limbica posterior lateralis R	38	Dorsal posterior cingulate	#6c7b8d
Amygdala L	39	Amygdala	#c089d0
Amygdala R	40	Amygdala	#c089d1
Insular cortex L	41	Sylvian L	#698839
Insular cortex R	42	Sylvian R	#d4c6bd
Lobus piriformis L	43	Amygdala	#c089d2
Lobus piriformis R	44	Amygdala	#c089d2
Thalamus L	45	Sylvian L	#698841
Thalamus R	46	Amygdala	#c089d2
Area septalis L	47	Medial prefrontal	#7ba5d14
Area septalis R	48	Medial prefrontal	#7ba5d15

ROI name	Code	Network	Hex code
G. presplenialis L	49	Dorsal posterior cingulate	#6c7b8d
G. presplenialis R	50	Dorsal posterior cingulate	#6c7b8d
FPSL_1 Fissura presylvian lateralis L	51	Medial cingulate	#cda64b
FPSL_2 Fissura presylvian lateralis R	52	Medial cingulate	#cda64b
G. cinguli L	53	Anterior cingulate	#99d78b
G. cinguli R	54	Anterior cingulate	#99d78b
G. precruciatus L	55	Somatomotor	#ced745
G. precruciatus R	56	Somatomotor	#ced746
GI_1 Genualis I L	57	Anterior cingulate	#99d78b
GI_2 Genualis I R	58	Anterior cingulate	#99d78b
GII_1 Genualis II L	59	Medial cingulate	#cda64b
GII_2 Genualis II R	60	Medial cingulate	#cda64b
LAL_1 Limbica anterior lateralis L	61	Medial cingulate	#cda64b
LAL_2 Limbica anterior lateralis R	62	Medial cingulate	#cda64b
LAV_1 Limbica anterior ventralis L	63	Medial cingulate	#cda64b
LAV_2 Limbica anterior ventralis R	64	Medial cingulate	#cda64b
LM_1 Limbica media L	65	Medial cingulate	#cda64b
LM_2 Limbica media R	66	Medial cingulate	#cda64b
XMII_1 Precruciata medialis I L	67	Anterior cingulate	#99d78b
XMII_2 Precruciata medialis I R	68	Anterior cingulate	#99d78b
G. compositus rostralis L	69	Somatomotor	#ced747
G. compositus rostralis R	70	Sylvian R	#d4c6bd
G. proreus L	71	Dorsal/lateral prefontal	#e57033
G. proreus R	72	Dorsal/lateral prefontal	#e57034
Nucleus caudatus L	73	Medial prefrontal	#7ba5d10
Nucleus caudatus R	74	Medial prefrontal	#7ba5d6
G. ectosylvius rostralis L	75	Sylvian R	#d4c6bd
G. ectosylvius rostralis R	76	Sylvian R	#d4c6bd
G. suprasylvius rostralis L	77	Somatomotor	#ced748
G. suprasylvius rostralis R	78	Somatomotor	#ced749
G. postcruciatus L	79	Somatomotor	#ced750
G. postcruciatus R	80	Somatomotor	#ced751
G. ectosylvius caudalis L	81	Sylvian L	#698842
G. ectosylvius caudalis R	82	Sylvian R	#d4c6bd

ROI name	Code	Network	Hex code
G. ectosylvius medius L	83	Sylvian L	#698843
G. ectosylvius medius R	84	Sylvian R	#d4c6bd
G. suprasylvius medius L	85	Sylvian R	#d4c6bd
G. suprasylvius medius R	86	Sylvian R	#d4c6bd
G. sylvius caudalis L	87	Sylvian L	#698844
G. sylvius caudalis R	88	Sylvian R	#d4c6bd
G. sylvius rostralis L	89	Sylvian L	#698845
G. sylvius rostralis R	90	Sylvian R	#d4c6bd
G. marginalis L	91	Visual	#79d8c11
G. marginalis R	92	Visual	#79d8c12
G. occipitalis L	93	Visual	#79d8c13
G. occipitalis R	94	Visual	#79d8c14
Hemispherium cerebelli L	95		NA
Hemispherium cerebelli R	96		NA
Vermis cerebelli	97		NA

Table S2. List of excluded brainstem and olfactory region ROIs

Bulbus olfactorius L
Bulbus olfactorius R
Commissura rostralis
Diencephalon
Hypophysis
Medulla oblongata
Medulla spinalis
Mesencephalon
Nervus opticus
Pedunculus olfactorius L
Pedunculus olfactorius
Pons
Tuberculum olfactorium L
Tuberculum olfactorium R
Hemispherium cerebelli L
Hemispherium cerebelli R
Vermis cerebelli
